# Dermatomyositis as a Paraneoplastic Syndrome of Metastatic Urothelial Carcinoma

**DOI:** 10.3390/curroncol33030176

**Published:** 2026-03-20

**Authors:** Amn Marwaha, Raman Sambhi, Ricardo Fernandes

**Affiliations:** 1 Schulich School of Medicine & Dentistry, Western University, London, ON N6A 5C1, Canada; amn.marwaha@lhsc.on.ca (A.M.); raman.sambhi@lhsc.on.ca (R.S.); 2 Division of Medical Oncology, Department of Oncology, Schulich School of Medicine & Dentistry, Western University, London, ON N6A 3K7, Canada; 3 Verspeeten Family Cancer Centre, London Health Sciences Centre, London, ON N6A 5W9, Canada

**Keywords:** dermatomyositis, metastatic urothelial carcinoma, paraneoplastic syndrome

## Abstract

A 65-year-old man with history of urothelial carcinoma of the bladder developed dermatomyositis at the time of metastatic disease progression. This temporal association supports a likely paraneoplastic mechanism and highlights the importance of reassessing for disease progression in patients with urothelial carcinoma who develop new-onset dermatomyositis. Early recognition may facilitate timely oncologic evaluation and multidisciplinary management.

## Simple Summary

Dermatomyositis is an inflammatory condition that causes muscle weakness and distinctive skin rashes, and in some people, it can be a warning sign of an underlying cancer or cancer recurrence. This report describes a man with a prior history of bladder cancer who developed new dermatomyositis symptoms during a period when his cancer initially appeared stable. As his muscle weakness and rash progressed, imaging soon confirmed that the bladder cancer had returned and spread. This close timing supports dermatomyositis as a likely cancer-related (paraneoplastic) condition in this setting and shows how easily early symptoms can be mistaken for medication reactions or other common problems in cancer care. Our case highlights the need for timely reassessment for cancer progression when dermatomyositis develops in patients with current or past bladder cancer, supported by coordinated care between oncology, rheumatology, and dermatology.

## 1. Introduction

Dermatomyositis (DM) is an idiopathic inflammatory myopathy characterized by proximal muscle weakness and cutaneous manifestations, such as Gottron’s papules and a heliotrope rash, that are pathognomonic for dermatomyositis [1]. Estimations of the incidence and prevalence of DM vary globally, with the incidence ranging from 0.8 to 4.0 per 100,000 adults and prevalence between 1.2 and 28.6 per 100,000 adults [2,3,4,5].

Paraneoplastic dermatomyositis is a notable subset of dermatomyositis cases as those with DM have a nearly 5-fold risk of developing malignancy [6,7]. Paraneoplastic dermatomyositis is most associated with a significant increase in risk of developing lymphomas and hematopoietic cancers, followed by lung, ovarian, and colon cancer [6]. The pathophysiology of paraneoplastic dermatomyositis is suspected to be the result of an autoimmune reaction where anti-tumor antibodies cross-react with antigens specific to tissues of the muscle and skin [8]. Despite unclear pathophysiology, the association between DM and malignancy is well established. In a study across two U.S. cohorts, 12% of patients developed malignancy within 5 years of DM, of which 40% were diagnosed within the one year [9]. As such, the presence of DM should prompt suspicion for underlying malignancy and the initiation of regular cancer screening as per the International Guideline for Idiopathic Inflammatory Myopathy-Associated Cancer Screening recommendations [10].

Dermatomyositis with urothelial carcinoma is incredibly rare, with few cases reported in the literature.

Although dermatomyositis is a recognized paraneoplastic syndrome, its occurrence in urothelial carcinoma is uncommon, and its temporal relationship with disease progression is not well characterized. Recognition of dermatomyositis in oncology patients is clinically important, as it may represent an early manifestation of malignancy or a marker of tumor recurrence.

Herein, we report a case of dermatomyositis developing in temporal association with metastatic progression of urothelial carcinoma. This case highlights dermatomyositis as a potential clinical indicator of disease recurrence and underscores the diagnostic challenge of distinguishing paraneoplastic manifestations from treatment-related adverse effects. Increased awareness of this association may facilitate earlier detection of progression and inform multidisciplinary management.

## 2. Case History

This case is of a 65-year-old male who presented to the Genitourinary Multidisciplinary Care Clinic (GUMCC) for evaluation of suspected recurrence of non-muscle-invasive bladder cancer, initially diagnosed in 2020. At that time, transurethral resection of bladder tumor (TURBT) showed a pathological non-muscle invasive high-grade papillary urothelial carcinoma, positive for carcinoma in situ and lymphovascular invasion. He underwent intra-vesicular Bacillus Calmette–Guérin treatment, and subsequent repeat resection demonstrated no evidence of residual malignancy. During routine surveillance imaging in December 2021, a 1.7 cm lytic lesion was identified in the right pubic ramus on computed tomography (CT) of the abdomen and pelvis. A CT scan of the chest revealed a 2 mm right-sided subpleural nodule, and a bone scan showed increased uptake in the sternum.

The patient was seen in consultation at the GUMCC in February 2022. He appeared well and functionally independent and reported maintaining an active lifestyle. Aside from occasional urinary urgency, he denied any other genitourinary, bowel or respiratory symptoms. He had no constitutional symptoms. He described a one-week history of intermittent, positional right-sided lower back pain that has since stabilized. On physical examination, the only notable finding was mild tenderness in the right groin, which was attributed to a musculoskeletal injury sustained while dismounting a ladder. There was no palpable lymphadenopathy or bony tenderness, and digital examination of the prostate was unremarkable.

The patient’s past medical history included chronic obstructive pulmonary disease (COPD), hypertension and a remote history of a motor vehicle accident. His family history was notable for colon cancer in his mother. He denied any alcohol use and was an active smoker with a 20-year history. He also reported moderate occupational exposure to chemical waste and byproducts during his career in the automotive industry.

## 3. Investigations

After review and discussion in the GUMCC, the patient underwent an interventional radiology-guided biopsy of the lytic bony lesion. Histopathological analysis confirmed high-grade carcinoma consistent with urothelial carcinoma origin. Repeat cystoscopy and staging CT scans showed interval enlargement of the pubic ramus lesion with extension into adjacent musculature as well as new asymmetry of the seminal vesicles. Bloodwork demonstrated normal chemistry panel with creatinine of 83 umol/L and calcium of 2.31 mmol/L. Hematology showed leukocytes 7.2 × 10^9^ U/L, hemoglobin 120 g/L, and platelets 341 × 10^9^ U/L. Liver enzymes were within the normal range and LDH was 188 U/L.

### 3.1. Examination

Prior to the initiation of systemic treatment, the patient was clinically well. His review of systems was unremarkable, apart from a single episode of nocturia and localized discomfort at the biopsy site. On physical examination, the patient was afebrile and exhibited normal respiratory, cardiac, abdominal, and neurologic findings.

### 3.2. Management/Treatment

Given the patient’s relatively young age and the absence of widespread metastatic disease, an aggressive treatment approach was recommended. He underwent concurrent chemoradiation treatment targeting the bladder, prostate, seminal vesicles and the lytic lesion in the pubic ramus. Radiation therapy was delivered at a dose of 55 Gy in 20 fractions over 4 weeks, with concurrent weekly cisplatin (40 mg/m^2^) infusions. The patient tolerated treatment well, with mild nocturia and fatigue. No dose modifications of interruptions were required.

A follow-up CT scan of the chest, abdomen and pelvis performed two months post-treatment revealed two subcentimeter right lower lobe subpleural nodules and interval enlargement of the right pubic ramus lesion, presumed to reflect post-radiation changes. In the absence of definitive progression, further systemic therapy was deferred, and the patient was transitioned to active surveillance.

Subsequent imaging demonstrated an acute, non-displaced pathological fracture of the right pubic ramus, secondary to progression of the osseous metastasis. He was referred to orthopedic surgery. Prophylactic fixation was offered; however, the patient declined the procedure. Systemic therapy and bone-modifying therapy were discussed. He was concerned with the possible side effects of the treatment and opted to proceed with zoledronic acid. In September 2022, he was initiated on intravenous zoledronic acid (4 mg every 28 days) for the prevention of skeletal-related events.

After the fourth cycle of zoledronic acid in December 2022, the patient developed what was believed to be an allergic hypersensitivity reaction. By the following day, he experienced diffuse body pain accompanied by bilateral facial and arm edema. Zoledronic acid was discontinued, and he was started on prednisone (25 mg oral daily for 7 days), diphenhydramine (50 mg oral daily), and extra-strength Tylenol (2 tablets b.i.d. PRN), resulting in subsequent improvement of the symptoms. The patient declined further treatment with zoledronic acid.

In January 2023, he developed purple bruising below both eyes. While the symptoms initially improved, he experienced worsening pain in his joints and muscles, particularly in his arms and shoulders. He also reported fatigue and shortness of breath on exertion. He denied symptoms suggestive of spinal cord compression or respiratory concerns including chest pain, cough, fevers, and hemoptysis. Repeat cystoscopy, chest, abdomen and pelvis CT scans, as well as bone scan, did not show any evidence of tumor recurrence or progression. The patient was referred to a neurologist with a focus on pain management to address the ongoing symptoms and was scheduled to return for surveillance imaging. At that time, dermatomyositis was not initially suspected, as symptoms were attributed to a possible hypersensitivity reaction to zoledronic acid and nonspecific musculoskeletal causes. However, with the subsequent progression of proximal muscle weakness, dysphagia, and the development of characteristic cutaneous findings, an inflammatory myopathy was increasingly considered in the differential diagnosis.

Prior to consultation with a neurologist, the patient sought care in the emergency department and from his general practitioner due to persistent pain and its impact on daily functioning. He reported a brief episode of hematuria, which resolved spontaneously. In April 2023, the patient developed progressive dysphagia to both solids and liquids, with the appearance of a violaceous, erythematous rash over the eyelids (heliotrope rash) and erythematous papules on the dorsal aspects of the metacarpophalangeal and interphalangeal joints (Gottron’s papules), along with significant unintentional weight loss. This was followed by worsening of myofascial pain involving the bilateral neck, shoulder girdle, and upper extremities, as well as progressive proximal muscle weakness, a predominant feature of dermatomyositis. Electromyography (EMG) demonstrated myopathic changes consistent with an inflammatory myopathy. Laboratory investigations revealed elevated levels of C-reactive protein, creatine kinase, and lactate dehydrogenase (Table 1). Given the presence of characteristic cutaneous findings, progressive proximal muscle weakness, and elevated muscle enzymes, dermatomyositis (DM) was strongly suspected, and a rheumatology consultation was carried out. Based on the clinical presentation, laboratory abnormalities, and EMG findings, a diagnosis of dermatomyositis was established. No skin or muscle biopsy was performed, and myositis-specific antibody testing was not carried out.

Immunosuppressive therapy with mycophenolate (360 mg tablets, po, b.i.d.) and prednisone (30 mg, po q.d.) was initiated, resulting in symptomatic improvement. However, subsequent surveillance CT imaging revealed disease progression with liver metastasis, retroperitoneal lymphadenopathy, and pulmonary nodules, consistent with metastasis spread. The temporal association between the onset of dermatomyositis symptoms and radiographic disease progression supported a likely paraneoplastic relationship.

In May 2023, the oncology team initiated systemic chemotherapy with cisplatin (70 mg/m^2^) and gemcitabine (1250 mg/m^2^, days 1 and 8 of a 21-day cycle). Prophylactic treatment for febrile neutropenia was given due to concurrent immunosuppressive therapy for dermatomyositis. The patient completed six cycles of platinum-based chemotherapy, which he generally tolerated well. Mild nausea and hematologic toxicity were noted, the latter requiring blood transfusion and subsequent chemotherapy dose reductions. Molecular testing for fibroblast growth factor receptor (FGFR) alterations was negative. Following completion of chemotherapy in August 2023, restaging CT imaging demonstrated stable disease, according to RECIST Criteria.

After multidisciplinary discussion with the patient and his rheumatologist, it was agreed to prioritize cancer control while managing the potential risk of a DM flare. In October 2023, avelumab (10 mg/kg) was initiated as a maintenance immunotherapy, with a plan to treat DM flares symptomatically if they occurred.

In December 2023, the patient developed joint pain, stiffness, fatigue, and muscle weakness, raising suspicion for a DM flare while on avelumab. Immunotherapy was held, and the patient was restarted on mycophenolate (500 mg, two tablets b.i.d.) and prednisone (20 mg daily). He was also briefly trialed on pregabalin for neuropathic symptoms, but this was discontinued after the onset of a rash, attributed either due to DM or medication side effects. The neuropathy eventually resolved.

**Figure 1:**
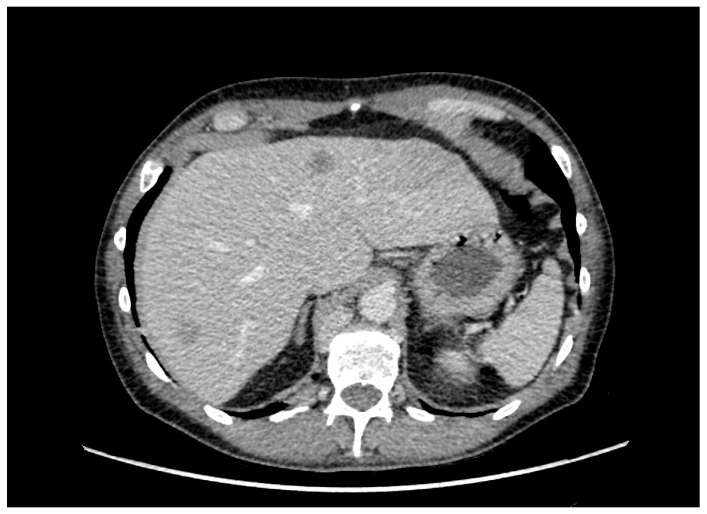

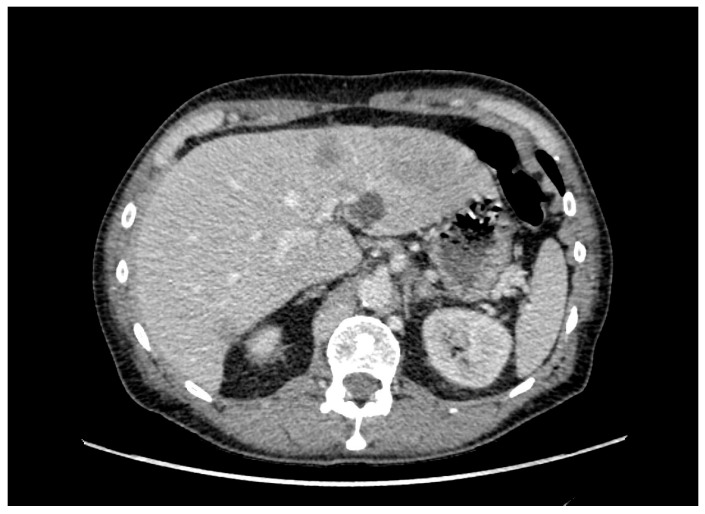
Imaging characteristics of metastatic progression of urothelial carcinoma on computed tomography (CT) of the abdomen.

Restaging CT imaging in January 2024 revealed radiographic disease progression (Figure 1), with accompanying clinical decline characterized by anorexia, fatigue, dyspnea on exertion, and worsening bone pain. The patient was started on third-line systemic therapy with an antibody–drug conjugate, enfortumab vedotin (EV) (1.25 mg/kg, intravenously on days 1, 8 and 15, every 28 days). Pain control was achieved with opioid therapy.

Shortly after, the patient presented to the emergency department with fever, diarrhea, and cognitive changes. He declined hospital admission and antibiotic treatment. However, symptoms subsided prior to the next scheduled EV dose.

By March 2024, after three cycles of EV, restaging pan-body CT scans demonstrated further disease progression with new metastasis to the liver and adrenal glands, as well as findings suspicious for intracranial involvement. The patient experienced rapid clinical deterioration, including frailty and poor performance status. MRI of the brain revealed imaging features concerning for intracranial hemorrhage. Given his declining condition and poor prognosis, radiation therapy was not pursued. EV was permanently discontinued, and the patient transitioned to comfort-focused palliative care. He died shortly thereafter.

## 4. Discussion

We present a case of dermatomyositis in a 65-year-old male with a history of urothelial carcinoma of the bladder. Dermatomyositis is a recognized paraneoplastic syndrome associated with solid malignancies; however, its occurrence in urothelial carcinoma remains rare. This case is notable for the temporal association between dermatomyositis onset and radiographic evidence of metastatic progression, supporting a likely paraneoplastic mechanism and highlighting its potential role as a clinical marker of disease recurrence.

Patients with DM have an elevated risk of malignancy compared to the general population [6]. A systematic review of paraneoplastic DM in bladder cancer found that only 13% of patients had bladder cancer diagnosed prior to the onset of DM [11]. In contrast to most reported cases in which dermatomyositis precedes or coincides with initial cancer diagnosis, our patient developed DM after definitive treatment and during a period of apparent disease control, with subsequent imaging confirming metastatic progression [11,12,13,14,15,16,17]. This temporal relationship suggests that dermatomyositis may serve as an early clinical indicator of occult disease progression in patients with urothelial carcinoma. Recognition of this association may facilitate earlier detection of recurrence and prompt reassessment of disease status.

The recent literature further supports the association between dermatomyositis and solid tumor progression, including urothelial carcinoma, although reports remain limited [18,19,20]. Compared with previously described cases, the delayed onset of dermatomyositis following initial cancer treatment in our patient reinforces its potential prognostic significance and highlights the need for ongoing malignancy surveillance in patients presenting with new inflammatory myopathies.

An important diagnostic consideration in this case was distinguishing drug-induced dermatomyositis from a paraneoplastic process. Although zoledronic acid (ZA) is generally well tolerated, rare cutaneous and inflammatory myopathy-like reactions have been reported [21,22,23,24]. Prior case reports describe dermatomyositis-like syndromes occurring within weeks of ZA administration, with symptom improvement following drug discontinuation and corticosteroid therapy [23,24]. In our patient, early symptoms including periorbital discoloration, myalgias, and edema developed following ZA administration and initially improved after treatment cessation, raising suspicion for a drug-related reaction. However, the subsequent progression to classical dermatomyositis features—including heliotrope rash, Gottron’s papules, proximal muscle weakness, and elevated muscle enzymes—occurred in parallel with radiographic evidence of metastatic disease progression. This temporal relationship suggests a paraneoplastic mechanism rather than a purely medication-induced phenomenon. These findings underscore the importance of maintaining a high index of suspicion for malignancy-associated dermatomyositis in oncology patients presenting with inflammatory myopathies, even when symptoms initially appear temporally related to pharmacologic therapy.

Management of dermatomyositis in patients with active malignancy presents significant clinical challenges, requiring careful coordination between symptom control and oncologic treatment priorities. In this case, systemic cancer therapy was initially prioritized over escalation of immunosuppressive therapy due to the urgency of controlling metastatic disease and the patient’s overall clinical status. However, progression of dermatomyositis symptoms significantly impaired functional status and quality of life, necessitating reinitiation of immunosuppressive therapy. This highlights the importance of individualized, multidisciplinary decision-making when managing paraneoplastic autoimmune syndromes, particularly when treatment strategies for malignancy and autoimmune disease may have competing risks and benefits.

Corticosteroids remain the recommended first-line therapy for dermatomyositis, with steroid-sparing agents such as mycophenolate mofetil commonly used in moderate-to-severe disease or for long-term management. In this patient, prednisone was initiated for rapid symptom control, with subsequent addition of mycophenolate to reduce corticosteroid exposure. Although immunosuppressive therapy in patients with active malignancy raises theoretical concerns regarding infection risk and tumor immune surveillance, treatment was necessary to address severe autoimmune manifestations and preserve functional status. This case illustrates the importance of balancing effective dermatomyositis treatment with ongoing cancer therapy and underscores the role of multidisciplinary collaboration between oncology and rheumatology teams.

Several limitations should be acknowledged. The patient received care across multiple institutions, which may have limited complete capture of all clinical documentation. In addition, no skin or muscle biopsy or myositis-specific antibody testing was performed, which may limit histopathologic confirmation and prognostic assessment. However, the presence of characteristic cutaneous findings, proximal muscle weakness, elevated muscle enzymes, and electromyographic findings fulfilled the 2017 EULAR/ACR classification criteria for idiopathic inflammatory myopathy [25], supporting the diagnosis of dermatomyositis. The absence of clinical photographs and histopathologic confirmation represents an inherent limitation of retrospective case reporting.

## 5. Conclusions

This case illustrates dermatomyositis as probably paraneoplastic manifestation and potential clinical marker of metastatic progression in urothelial carcinoma. New-onset dermatomyositis in patients with current or prior history of bladder cancer should prompt timely reassessment for disease recurrence or progression, even when prior imaging suggests stability. Clinicians should carefully distinguish between drug-induced and paraneoplastic etiologies, as misattribution to treatment-related toxicity may delay appropriate oncologic evaluation. Management requires a multidisciplinary approach involving oncology, rheumatology, and dermatology to balance effective immunosuppressive therapy with ongoing cancer treatment. Increased awareness of this association may facilitate earlier detection of progression and improve patient-centered clinical decision-making.

## Figures and Tables

**Table 1:** Bloodwork Prior to Dermatomyositis Diagnosis.

Name	Result	Normal Range	Units
Erythrocytes	3.6	4.0–11.0	×10^12^/L
Leukocytes	**12.0**	4.0–11.0	×10^9^/L
Hemoglobin	**105**	135–175	g/L
Platelets	**452**	150–400	×10^9^/L
Creatine kinase	**2246**	44–275	U/L
Lactate dehydrogenase	**392**	110–230	U/L
ALT	40	<50	U/L
C reactive protein	**5.4**	<5	mg/L
Erythrocyte sedimentation rate	25	2–30	mm/hr
IgG	10.97	6.00–16.00	g/L
IgA	1.74	0.54–4.17	g/L
IgM	1.34	0.3–2.3	g/L
Complement C3	1.16	0.9–1.80	g/L
Complement C4	0.38	0.15–0.53	g/L
dsDNA antibody	<1	<5	IU/mL
Nuclear antibody	Negative	Negative	-
Extractable nuclear antibody	Negative	Negative	-

The bold refers to abnormal results.

## Data Availability

The data that support the findings of this study are available on request from the corresponding author. The data are not publicly available due to privacy or ethical restrictions.
